# Five‐year observational study of Internet‐delivered cognitive behavioural pain management when offered as routine care by an online therapy clinic

**DOI:** 10.1002/ejp.1866

**Published:** 2021-10-12

**Authors:** Heather D. Hadjistavropoulos, Vanessa Peynenburg, David Thiessen, Luke H. Schneider, Marcie Nugent, Andrew Wilhelms, Eyal Karin, Nickolai Titov, Blake F. Dear

**Affiliations:** ^1^ Department of Psychology University of Regina Regina Saskatchewan Canada; ^2^ Department of Mathematics & Statistics University of Regina Saskatchewan Canada; ^3^ Department of Psychology MindSpot Clinic and eCentreClinic Macquarie University Sydney Australia

## Abstract

**Background:**

Internet‐delivered cognitive behavioural pain management programmes (PMPs) are effective, but less is known about their use outside of research trials. Five years of data from offering the Internet‐delivered cognitive behavioural PMP in an online therapy clinic was examined to assess effectiveness, acceptability and predictors of outcomes.

**Methods:**

Patients (*N* = 293) were offered a previously validated 8‐week Internet‐delivered cognitive behavioural PMP and administered measures at pre‐treatment, post‐treatment and 3 months.

**Results:**

There was growth in demand for an Internet‐delivered cognitive behavioural PMP over time (*n* = 64 first year to *n* = 133 fifth year). Moderate‐to‐large improvements on depression (post‐treatment 35% reduction; 3‐month 41% reduction) and anxiety (post‐treatment 37% reduction; 3‐month 41% reduction), and small‐to‐moderate improvements on disability (post‐treatment 19% reduction; 3‐month 20% reduction) were found. Lesson completion and satisfaction were high. Lower pain acceptance, lower pain self‐efficacy and higher pain intensity were associated with lower improvements on depression, anxiety and disability.

**Conclusions:**

This longitudinal observational study provides support for Internet‐delivered cognitive behavioural PMPs when offered as routine care by an online therapy clinic.

**Significance:**

This 5‐year observational study provides support for Internet‐delivered cognitive behavioural pain management programs (PMPs) offered as routine care in an online therapy clinic. Interest in the service grew over 5 years. Outcomes, engagement and satisfaction were strong. Higher pain acceptance, pain self‐efficacy and lower pain severity were associated with greater post‐treatment improvements on depression, anxiety and disability.

**What's already known?**

- Internet‐delivered cognitive behavioural pain management programmes (PMPs) are effective in research trials.
- Internet‐delivered cognitive behavioural PMPs result in significant improvements in depression, anxiety and disability.

**What does this study add?**

- This 5‐year observational study shows growth in demand for an Internet‐delivered cognitive behavioural PMP when offered as routine care in an online clinic.
- Outcomes were strong when an Internet‐delivered cognitive behavioural PMP was delivered in a routine online clinic, and previous clinical trial outcomes were replicated.
- Higher baseline pain acceptance and pain self‐efficacy, and lower pain severity were associated with greater post‐treatment improvements.

## 1 INTRODUCTION

Nearly one in five Canadian adults experience chronic pain at any given time, with about one‐half of those reporting these symptoms for 10 years or more, and one‐third describing their pain as severe (Schopflocher et al., 2011). Patients with chronic pain experience elevated levels of psychological distress (Reitsma et al., 2011), contributing to increased healthcare costs (Hogan et al., 2016). Cognitive behavioural pain management programmes (PMPs) are effective in reducing co‐morbid psychological distress (Williams et al., 2012), but are costly and not widely available (Ehde et al., 2014).

Internet‐delivered cognitive behavioural PMPs have significant potential for increasing access to effective pain management (Eccleston et al., 2020). In Internet‐delivered cognitive behavioural programmes, patients complete structured online materials that parallel face‐to‐face treatment content, with the period of treatment in many programmes ranging from 3 weeks to 11 months (Buhrman et al., 2016; Eccleston et al., 2014). The majority of programmes are offered with therapist support provided by a research assistant, graduate student in psychology or psychologist (Buhrman et al., 2016) and most are offered through telephone and or secure messaging (e.g. Dear et al., 2015). The *Pain Course* is one such programme that has been studied extensively in randomized controlled trials (RCTs), under various conditions, including weekly contact with a therapist, optional contact with a therapist or without therapist contact (Dear et al., 2015, 2018). Regardless of conditions, patients report clinically meaningful improvements in depression, anxiety, disability and pain intensity compared with a waitlist control group (Dear et al., 2013, 2015, 2018). Thus, there is now a growing body of evidence supporting the potential of Internet‐delivered cognitive behavioural PMPs as another way of providing care to people with chronic pain.

Despite the encouraging evidence from RCTs, there have been no large‐scale reports of Internet‐delivered cognitive behavioural PMPs when offered on a routine basis (Buhrman et al., 2016). Evaluations in such real‐world settings are, however, essential because patients are known to present with greater symptom severity, complexity and diversity than is often observed among patients in early phase RCTs (Gibbons et al., 2013). Such evaluations can therefore provide valuable data to clinicians and services about the types of patients who are and are not likely to benefit from new interventions, potentially informing service planning and care delivery.

This study examines the effectiveness of an Internet‐delivered cognitive behavioural PMP when offered as part of routine care in an online therapy clinic over 5 years in terms of acceptability and outcomes. Furthermore, in this study, we examined baseline demographic, clinical and psychological predictors of depression, anxiety and disability. It was hypothesized that patients receiving an Internet‐delivered cognitive behavioural PMP would be engaged with and satisfied with treatment, and report significant improvements on primary (depression, anxiety, disability) and secondary (pain severity, pain self‐efficacy, fear of movement, chronic pain acceptance) measures. Predictors of treatment were considered exploratory, given limited past research on the topic.

## 2 METHODS

### 2.1 Context

Saskatchewan is located in west‐Central Canada and has a population of ~1.1 million, with most residents identifying as Caucasian (Statistics Canada, 2017a). Approximately half the population lives in two large cities over 250,000 residents, and other residents live in small cities, towns or rural and remote areas (Statistics Canada, 2017b). Throughout Canada, medically necessary medical office and hospital care are publicly funded (Marchildon et al., 2014); in addition, the government also funds some, but not all, mental healthcare. The Online Therapy Unit (www.onlinetherapyuser.ca) has been funded by the province to provide free Internet‐delivered cognitive behaviour therapy to adult residents of Saskatchewan since 2015 (see Hadjistavropoulos et al., 2021 for more information). The unit offers an Internet‐delivered cognitive behavioural PMP, the *Pain Course*, for people with chronic pain as one of its free services.

### 2.2 Patients

All patients who completed the online suitability assessment for the *Pain Course* between May 3, 2015 and February 24, 2020 were included in this study. In total, 512 individuals completed the online suitability assessment, with 490 of the individuals found to be suitable for the course. The most common reason for non‐suitability was living outside of Saskatchewan (*n* = 10), followed by limited access to a personal computer or Internet (*n* = 8). Of the 490 individuals deemed suitable, 415 completed a follow‐up telephone interview to further assess programme suitability (75 patients could not be reached). Based on the online and telephone suitability assessment, a total of 311 patients were accepted into the *Pain Course*, with 293 of those patients starting the intervention.

Table 1 shows background characteristics of the sample. An examination of the sample revealed that patients learned about the *Pain Course* via physicians/medical professionals (41.0%; *n* = 120), other referrals (i.e. mental health professionals, bariatric clinic referral, and referral from a friend, family member or employer; 38.9%; *n* = 114) and other sources (i.e. printed posters or cards, media or online sources; 20.1%, *n* = 59).

**TABLE 1:** Patient characteristics

	*N* = 293	
	*n*	%
Age		
Mean (SD)	44.25 (13.68)	—
Range	18–82	—
Gender		
Male	78	26.6
Female	215	73.4
Marital status		
Single/never married	59	20.1
Married	145	49.5
Living with partner	44	15.0
Separated	15	5.1
Divorced	24	8.2
Widowed	4	1.4
Undisclosed	2	0.7
Education		
Less than high school	11	3.8
High school diploma	63	21.5
Post high school certificate/diploma	88	30.0
Some university	49	16.7
University undergraduate degree	55	18.8
University professional degree (e.g. MD)	9	3.1
University graduate degree (e.g. MA, PhD)	18	6.1
Employment status		
Employed part‐time/full time	108	36.9
Unemployed	30	10.2
Homemaker	15	5.1
Student	8	2.7
Retired	28	9.6
Short‐term disability	32	10.9
Long term disability	72	24.6
Ethnicity		
Caucasian	270	92.2
Indigenous/Metis/Inuit	7	2.4
Mixed ethnicity	7	2.4
Other	8	2.7
Undisclosed	1	0.3
Location		
Large city (over 200,000)	123	42.0
Small city (10k–200k)	77	26.3
Town/village	68	23.2
Farm	25	8.5
Duration of pain symptoms (years)		
Mean (SD)	6.54 (7.96)	—
Range	0.25–45.00	—
Pain location(s)		
Upper back/middle back/lower back	209	71.3
Hip/pelvis/leg/foot	191	65.2
Shoulder/arm/hand	149	50.9
Head/face	96	32.8
Other	76	25.9
Average number of pain sites (SD)	4.45 (3.10)	—
Mental health characteristics		
Infrequent use of some form of mental health treatment	154	52.6
Pre‐treatment GAD−7 ≥8	196	66.9
Pre‐treatment PHQ−9 ≥ 10	205	70.0
Referral source		
Physician/medical professional referral	120	41.0
Other referral (mental health professionals, bariatric clinic referral or family member/friend/employer)	114	38.9
Other source (printed materials, media or online sources)	59	20.1

To be suitable for the *Pain Course*, prospective patients had to: (1) self‐report chronic pain for >3 months and report past contact with a physician about their pain; (2) self‐report symptoms of low mood or worry (but not be at high risk of suicide; as assessed during the telephone interview); (3) reside within Saskatchewan for the 8‐week course of treatment; (4) be at least 18 years of age; (5) be willing to provide a medical contact in the event of an emergency; (6) be comfortable using the Internet and (7) not be receiving face‐to‐face therapy more than twice per month. Of note, only a small subset of patients (6.5%, *n* = 19/293) reported up to two mental health visits per month. Eligible patients were enrolled in the course immediately after the telephone screening and did not receive remuneration for participating.

### 2.3 Design and measures

The study received research ethics approval through the University of Regina Research Ethics Board and the trial was registered as an observational trial (ISRCTN15509834) to assess the effectiveness and acceptability of the *Pain Course*. In past analyses of Internet‐delivered therapy in routine care, a time frame ranging from 30 months (e.g. Titov et al., 2017) to 6 years (e.g. Hadjistavropoulos et al., 2021) has been used to evaluate services over time. For the *Pain Course*, a period of 5 years was chosen to allow for observations about growth over time and by this period of time approximately 300 patients had been assessed for suitability, providing a sufficient sample for the planned analyses. Patients completed outcome measures online at pre‐treatment, post‐treatment and 3‐month follow‐up. As the study was conducted over 5 years, some changes occurred in how some questions were asked which limited the amount of data related to health service use, medication use and disability (see below). During the first 2 years of offering the course, if patients did not complete follow‐up measures, an attempt was made to collect primary (i.e. depression, anxiety and disability), but not secondary outcome measures (i.e. pain severity, pain self‐efficacy, kinesiophobia, pain acceptance and health service use) via telephone or survey link sent via email. Consequently, we have limited secondary measures at 3‐month follow‐up for participants during the first 2 years of the trial.

### 2.4 Primary measures

#### 2.4.1 Generalized Anxiety Disorder 7‐item (GAD‐7)

The GAD‐7 is a 7‐item psychometrically sound self‐report measure of generalized anxiety, with each item rated on a scale of 0 (*not at all*) to 3 (*nearly every day)*. Scores ≥8 can identify probable cases of generalized anxiety disorder with adequate specificity and scores <5 represent minimal symptoms of anxiety (Spitzer et al., 2006). In the present study, Cronbach's α at pre‐treatment, post‐treatment and follow‐up ranged from 0.89 to 0.92.

#### 2.4.2 Patient Health Questionnaire 9‐item (PHQ‐9)

The PHQ‐9 is a 9‐item psychometrically sound self‐report measure of symptoms of depression, with items rated from 0 (*not at all*) to 3 (*nearly every day*). A score ≥10 has been used as a cut‐off for identifying individuals who are likely to meet the criteria for major depressive disorder (Kroenke et al., 2001). Scores <5 represent minimal symptoms of depression (Kroenke et al., 2001). In this study, Cronbach's α ranged from 0.85 to 0.90 across the three administrations.

#### 2.4.3 Roland Morris Disability Questionnaire (RMDQ)

The RMDQ includes 24 yes/no items assessing disability. Consistent with previous research, the word ‘back pain’ was replaced with ‘pain’ to make it applicable to diverse chronic pain conditions (e.g. Dear et al., 2013; Hadjistavropoulos et al., 2018). Scores >14 have been used to identify clinically significant pain‐related disability (Dear et al., 2015), while scores ≤7 have been used to identify those with minimal disability (Dear et al., 2016). In the present study, Cronbach's α ranged from 0.87 to 0.90 across the three administrations. Of note, a change in software for collecting outcome measures resulted in an administration error whereby this measure was not given to the last 139 participants Roland & Morris, 1983.

### 2.5 Secondary measures

The following measures were selected to be consistent with past research on the *Pain Course* (Dear et al., 2013, 2015; Friesen et al., 2017; Hadjistavropoulos et al., 2018).

#### 2.5.1 The Brief Pain Inventory (BPI)

The BPI is a validated measure used to assess the intensity and interference of pain in an individual's daily life. Only the items asking about the intensity of current pain, average pain and least and worst pain in the last 24 h were included in this study. Items are rated from 0 (*no pain*) to 10 (*pain as bad as you can imagine*). The four BPI pain severity items were combined to create a mean composite score. Cronbach's α in the current study ranged from 0.86 to 0.92 across administrations Cleeland & Ryan, 1994.

#### 2.5.2 Pain Self‐Efficacy Questionnaire (PSEQ)

The PSEQ is a 10‐item measure that assesses patient beliefs about their ability to complete daily tasks while experiencing pain. Each item is rated on a 7‐point scale, with higher scores indicating greater pain‐related self‐efficacy. The PSEQ has good internal consistency and test–retest reliability (Nicholas, 2007). Within the current study, Cronbach's α ranged from 0.91 to 0.94 across administrations.

#### 2.5.3 TAMPA Scale of Kinesiophobia (TSK)

The TSK includes 17 items assessing fear of movement and re‐injury. Each item is rated on a 4‐point scale, with higher scores indicating greater fear of movement and re‐injury. The TSK has strong psychometric properties (Swinkels‐Meewisse et al., 2003). Cronbach's α in this study ranged from 0.78 to 0.84 across administrations Burwinkle et al., 2005.

#### 2.5.4 Chronic Pain Acceptance Questionnaire 8‐Item (CPAQ‐8)

The CPAQ‐8 includes eight items that assess patients’ acceptance and willingness to experience pain, with higher scores indicating greater acceptance and willingness. The CPAQ‐8 has strong psychometric properties (Fish et al., 2010). Cronbach's α in this study ranged from 0.83 to 0.84 across administrations.

#### 2.5.5 Health service and medication use

Over the 5‐year period, there was variability in questions that assessed patients’ use of various health professionals (i.e. general practitioner/nurse, psychiatrist, psychologist/counsellor, medical specialist or emergency service) and medication use. Consequently, in this study, we report data for a subsample of patients enrolled from January 2018 onwards, specifically reporting whether patients had seen certain medical professionals or taken prescription medication for pain or mental health in the 8 weeks before completing pre‐treatment and 3‐month follow‐up measures.

### 2.6 Treatment acceptability

#### 2.6.1 Programme engagement

As tracked by the website, programme engagement was explored by examining the number of lessons completed, number of messages sent by the patient to the therapist, and total times therapists either emailed the patient and or recorded a phone call to patient.

#### 2.6.2 Treatment satisfaction

At post‐treatment, consistent with past Internet‐delivered cognitive behavioural PMPs (e.g. Dear et al., 2013, 2015; Friesen et al., 2017; Hadjistavropoulos et al., 2018), patients were asked whether the course was worth their time and whether they would recommend it to a friend using a yes/no response option.

### 2.7 Treatment programme

The *Pain Course* (Dear et al., 2013, 2015, 2018) was developed by the eCentreClinic at Macquarie University (Australia) and is now offered by the MindSpot Clinic (https://mindspot.org/au). It was licensed for use to the Online Therapy Unit. It consists of five lessons that are released over the span of 8 weeks, with each lesson consisting of a slideshow, downloadable guide and case stories and examples. The five lessons, which patients are encouraged to work on for 1 to 2 weeks each, are intended to help patients understand chronic pain and manage its impacts on their day‐to‐day lives and emotional wellbeing. The Course includes psychoeducation on: (1) the cognitive behavioural model of pain; (2) cognitive restructuring; (3) controlled breathing and pleasant activity scheduling; (4) graded exposure and pacing and (5) relapse prevention and goal setting. Lesson 1 is available immediately after clients are enrolled. Subsequent lessons are released based on elapsed time and the patient having accessed the previous lesson; specifically, lessons 1, 2, 3, 4 and 5 are available at the beginning of weeks 1, 2, 4, 5 and 7. Patients can download additional resources that address topics or concerns that are common among individuals with chronic pain (e.g. assertive communication, attention, pain management, pleasant activities, sleep, managing beliefs, managing panic, problem solving, working with health professionals and emergency contact information). In addition to emails and telephone calls, patients received weekly standardized automated messages to remind them of upcoming course content.

### 2.8 Therapist support

The purpose of therapist support was to summarize content, encourage practice of skills, reinforce progress and answer patient questions. Additionally, therapists normalized challenges in learning skills and managing symptoms. The therapists did not introduce any new therapeutic skills. Several different models of providing therapist support were used across the 5‐year time frame, reflecting therapist and patient experiences and preferences. Between May 2015 and January 2017, 74 patients received this support primarily during a telephone call. If therapists could not reach patients by telephone, then therapists sent a brief email containing the same information that would have been relayed in the telephone call. Given that therapists often could not reach and speak to patients by telephone after multiple attempts and were often sending emails, patients accepted between February 2017 and January 2018 were given the choice of telephone (*n* = 24) or email contact (*n* = 45). If patients selected a preference for email contact, patients were encouraged to write to their therapist during the week. The therapist then responded to all messages on a designated day once per week. Other than differences in the method of offering support, the content and amount of time spent on providing support per patient was the same (e.g. 10 to 15 min). When email support was offered, telephone calls were made only if clinically indicated (e.g. high suicide risk, concern about patient understanding). Subsequently between January 2018 and February 2020, given the strong patient and therapist preference for email contact, patients were consistently assigned to receive email support (*n* = 150) with phone calls only made when clinically indicated as described above. All emails were exchanged on the secure messaging system on the Online Therapy Unit platform.

In total, five therapists provided support with training as follows: one doctoral‐level clinical psychology graduate student (*n* = 66), two Master’s level registered social workers (*n* = 37 and 139), one Master's level certified counsellor (*n* = 52) and one Bachelor’s level registered social worker (*n* = 16). All clinicians were trained in Internet‐delivered therapy, which included attending a 1‐day workshop. Subsequently, all messages sent to patients were reviewed by a supervisor over the course of several months. After the supervisor approved independent practice, therapists attended regular Online Therapy Unit meetings and received supervision as requested. Previous studies have not found significant differences in acceptability or outcomes when support is offered by either graduate student practitioners or registered healthcare professionals (e.g. Johnston et al., 2011).

### 2.9 Analyses

Analyses were conducted using SPSS version 26 and R version 4.0.4. Descriptive statistics of the sample were calculated to describe the pre‐treatment characteristics of the sample. Next, changes in primary and secondary measures from pre‐treatment to post‐treatment to follow‐up were modelled using generalized estimating equations (GEE). GEE models estimate changes in a population mean while adjusting for correlated responses within individuals (Hardin & Hilbe, 2013; Hubbard et al., 2010). All GEE models used a Gamma distribution with log link to model proportional changes and accommodate skewed response distributions. Effectiveness of treatment was evaluated by examining estimated percentage changes from pre‐treatment to post‐treatment and follow‐up and tests on the parameters from the GEE models. We also calculated Cohen's *d*_unb_ effect sizes based on the estimated mean and standard deviations at post‐treatment and follow‐up (Cumming, 2012). These effect sizes assume linear, rather than proportional, changes from pre‐treatment. This represents another way to measure change compared to percentage change and is included as it also allows us to evaluate if our conclusions are similar using different methods of assessing outcomes.

Missing outcome measures were replaced using predictive mean matching multiple imputation before fitting the GEEs. To specify the imputation models, we examined patterns of data missingness. As in previous studies (Hadjistavropoulos et al., 2020; Karin et al., 2018), lesson completion was associated with increased completion of post‐treatment and 3‐month follow‐up measures (*p's* <.001). Of note, completion of secondary measures at 3‐month follow‐up was limited at the beginning of this study but improved over the first 2 years. Imputations controlled for lesson completion, method of support (in the event receiving support primarily via telephone or email impacted outcomes), any observed values of that measure for that patient at pre‐treatment, post‐treatment, or follow‐up and interactions.

We also examined the proportion of the sample that experienced a clinically significant recovery or deterioration at post‐treatment on the PHQ‐9, GAD‐7 or RMDQ using complete cases rather than imputed data. Due to the small number of patients deteriorating, appropriate imputation models could not be found for deterioration, therefore we only consider complete cases. To avoid modelling spurious changes in patients with minimal symptoms, we excluded patients with pre‐treatment PHQ‐9 ≤ 4, GAD‐7 ≤ 4 or RMDQ ≤ 7 in calculations for that measure. Patients who were not administered the RMDQ were also excluded from the RMDQ calculations. Consistent with previous research and recommendations (Dear et al., 2016; Hadjistavropoulos et al., 2020), clinical response was defined as above a 30% reduction in the PHQ‐9, GAD‐7 or RMDQ, respectively, from pre‐treatment to post‐treatment or follow‐up. Deterioration was defined as a 30% increase in symptoms over the same time. As a partial control for number of analyses performed, in all analyses described above, results were considered significant if *p* was ≤0.01.

To examine whether any patient characteristics were associated with treatment response, we used a collection of univariate logistic regression models. As above, clinical response was defined as a 30% reduction in the PHQ‐9, GAD‐7 or RMDQ at post‐treatment and we excluded patients with pre‐treatment PHQ‐9 or GAD‐7 scores ≤4 or RMDQ scores ≤7. A broad range of available pre‐treatment demographic and clinical variables was examined as predictors. Patients with missing post‐treatment measures had their improvement indicator variables multiply imputed controlling for lesson completion, method of support (to control for this methodological change over time) and the independent variable used in that model. Consistent with previous research (Dear et al., 2016), a liberal significance level of 0.1 was used for these logistic regressions to detect any important trends in predicting clinical outcomes. To evaluate the explanatory power of these models we calculated the coefficient of discrimination, D, the mean predicted probability of response among patients who did respond minus the mean predicted probability of response among patients who did not respond (Tjur, 2009).

## 3 RESULTS

### 3.1 Baseline

Of the 311 patients accepted into the *Pain Course*, 18 patients did not start the *Pain Course*, leaving 293 patients eligible for analysis. Table 1 includes demographics, pain history and mental health characteristics at pre‐treatment. The mean age of patients was 44.25 years (SD = 13.68) and the majority of patients were female (73.4%, *n* = 215), married or living with their partner (64.5%, *n* = 189), had at least a high school diploma (96.2%, *n* = 282), and were Caucasian (92.2%, *n* = 270). Only 42.0% (*n* = 123) resided in a large city. A significant number of patients were on short‐term or long‐term disability, with a wide range in the duration of pain symptoms (*M* = 6.54 years, SD = 7.96). On average, patients reported 4.45 pain sites (SD = 3.10), with upper back/middle back/lower back pain reported by the largest number of patients (71.3%, *n* = 209). At pre‐treatment, most patients’ scores were in the clinical range for anxiety (66.9%, *n* = 196) and depression (70.0%, *n* = 205), and 52.6% (*n* = 154) had infrequent use of some form of mental health treatment. Prescription medication and health service use at pre‐treatment for patients enrolled after January 2018 (*n* = 150) are summarized in Table 2. The majority of patients were taking medication for their pain or mental health (82.0%, *n* = 123) and had seen a GP/nurse/medical specialist in the 8 weeks prior to treatment (54.0%, *n* = 81). A smaller subset had seen a psychiatrist (21.3%, *n* = 32) or a psychologist/counsellor in the 8 weeks prior to treatment (28.7%, *n* = 43).

**TABLE 2:** Medication and health service use at pre‐treatment and 3‐month follow‐up^a^

	Pre‐treatment (*n* = 150)		3‐month follow‐up (*n* = 108)		Within‐group difference from pre‐treatment to 3‐month follow‐up
	*n*	%	*n*	%	Significance^b^
Prescription medication use for pain and mental health	123	82.0	61	57.0	*ꭙ2* (1, *n* = 107) = 20.83, *p* < 0.001
Healthcare service use					
GP/nurse/medical specialist	81	54.0	49	45.4	*ꭙ2* (1, *n* = 108) = 4.65, *p* = 0.03
Psychiatrist	32	21.3	19	17.6	*ꭙ2* (1, *n* = 108) = 0, *p* = 1.0
Psychologist/counsellor	43	28.7	36	33.3	*ꭙ2* (1, *n* = 108) = 0.11, *p* = 0.75

^a^ Includes data from patients enrolled from January 2018 onward.

^b^ McNemar test (Mcnemar, 1947) was used to examine differences in the proportion of patients responding ‘yes’ from pre‐treatment to 3‐month follow‐up. Results were considered significant at *p* ≤ 0.01.

### 3.2 Programme engagement and attrition

Table 3 summarizes programme engagement variables with 79.5% of patients completing all five lessons and patients logging into the intervention website on average 21.36 (SD = 13.44) times over the 8‐week period. Patients on average sent 3.76 (SD = 3.34) emails to therapists and therapists were in contact with patients a total of 10.10 (SD = 2.23) times either by email and or phone. As a further measure of engagement, 78.2% of patients completed primary measures at post‐treatment and 67.9% at 3‐month follow‐up.

**TABLE 3:** Programme engagement

	(*N* = 293)	
	*n*	%
Completion of 5 Lessons	233	79.5
Completion of post‐treatment primary measures^a^	229	78.2
Completion of 3‐month follow‐up primary measures^a^	199	67.9
Completion of post‐treatment secondary measures^b^	220	75.1
Completion of 3‐month follow‐up secondary measures^b^	129	44.0
Mean number of log‐ins (SD)	21.36 (13.44)	−
Mean number of times therapist contacted patient by phone and or email	10.10 (2.23)	−
Indicated course was worth time^c^	203	96.2%
Feel confident recommending course to a friend^c^	207	98.1%

^a^ Primary measures included the Patient Health Questionnaire‐9 (PHQ‐9) and General Anxiety Disorder‐7 (GAD‐7) questionnaires; the RMDQ was not used in this calculation due to an administration error that resulted in the measure not being given to the last 139 patients.

^b^ Secondary measures included the Brief Pain Inventory (BPI), Pain Self‐Efficacy Questionnaire (PSEQ), TAMPA Scale of Kinesiophobia (TSK) and Chronic Pain Acceptance Questionnaire 8‐item (CPAQ‐8). Percentages for those who indicated the course was worth their time and felt confident recommending the course to a friend are based on valid responses (*n* = 203 and *n* = 207 respectively), and do not include those who did respond to the post‐treatment questionnaire. Results were considered significant at *p* ≤ 0.01.

^c^ Percentages are based on the number of patients who completed the treatment satisfaction questions (*n* = 211).

### 3.3 Treatment satisfaction

As seen in Table 3, 96.2% (*n* = 203) of patients who completed post‐treatment questionnaires reported that the *Pain Course* was worth their time and 98.1% (*n* = 207) that they would feel confident recommending the course to a friend.

### 3.4 Primary outcome measures

Estimated means, standard deviations, percentage reductions and Cohen's *d*_unb_ effect sizes for all measures are presented in Table 4. There were significant time effects on all primary measures from pre‐treatment to post‐treatment and follow‐up (PHQ‐9: *F*_(2264)_ = 66.2, *p* < 0.001; GAD‐7: *F*_(2320)_ = 55.9, *p *< 0.001; RMDQ: *F*_(2182)_ = 13.5, *p* < 0.001). The reductions on the PHQ‐9 and GAD‐7 were moderate at post‐treatment and large at 3‐month follow‐up. Reductions on the RMDQ were moderate both at post‐treatment and at 3‐month follow‐up.

**TABLE 4:** Estimated marginal means, standard deviations, percentage changes, effect sizes (Cohen's d_unb_), 95% confidence intervals for primary and secondary outcomes (pooled imputations under MAR assumption) *p* ≤ 0.01

	Estimated marginal means			Percentage changes from pre‐treatment		Within‐group effect sizes from pre‐treatment	
	pre‐treatment	post‐treatment	3‐month follow‐up	to post‐treatment	to 3‐month follow‐up	to post‐treatment	to 3‐month follow‐up
Primary outcomes							
PHQ−9 (*N* = 293)	12.82 (5.79)	8.27 (6.16)	7.60 (5.36)	0.35 [0.28, 0.43]	0.41 [0.35, 0.46]	0.76 [0.59, 0.93]	0.94 [0.76, 1.11]
GAD−7 (*N* = 293)	10.60 (5.52)	6.64 (5.35)	6.21 (5.28)	0.37 [0.30, 0.44]	0.41 [0.35, 0.48]	0.73 [0.56, 0.90]	0.81 [0.64, 0.98]
RMDQ (*N* = 154)	13.86 (5.40)	11.20 (6.16)	11.09 (5.90)	0.19 [0.12, 0.27]	0.20 [0.11, 0.29]	0.46 [0.23, 0.69]	0.49 [0.26, 0.72]
Secondary outcomes							
BPI‐Severity (*N* = 293)	5.20 (1.55)	4.57 (2.02)	4.73 (1.98)	0.12 [0.07, 0.18]	0.09 [0.03, 0.15]	0.35 [0.19, 0.52]	0.27 [0.10, 0.43]
CPAQ−8 (*N* = 293)	19.57 (8.11)	23.82 (7.91)	24.69 (8.63)	−0.22 [−0.28, −0.16]	−0.26 [−0.33, −0.19]	−0.53 [−0.70, −0.37]	−0.61 [−0.78, −0.44]
PSEQ (*N* = 293)	27.06 (12.61)	35.98 (13.23)	34.42 (13.97)	−0.33 [−0.40, −0.26]	−0.27 [−0.35, −0.19]	−0.69 [−0.86, −0.52]	−0.55 [−0.72, −0.39]
TSK (*N* = 293)	40.79 (6.31)	36.37 (6.75)	35.89 (6.81)	0.11 [0.09, 0.13]	0.12 [0.09, 0.15]	0.68 [0.51, 0.84]	0.75 [0.58, 0.91]

Note

PHQ‐9, Patient Health Questionnaire‐9; GAD‐7, Generalized Anxiety Disorder‐7; RMDQ, Roland Morris Disability Measure; BPI‐Severity, Brief Pain Inventory; CPAQ‐8, Chronic Pain Acceptance Questionnaire 8‐item; PSEQ, Pain Self‐Efficacy Questionnaire; TSK, TAMPA Scale of Kinesiophobia; Standard deviations are shown in rounded parentheses for the estimated means; 95% confidence intervals are shown in square parentheses for the percentage changes and effect sizes.

Rates of clinically significant improvement and deterioration are presented in Table 5. At post‐treatment, 59% of the sample experienced clinically significant improvement on the PHQ‐9, 60% on the GAD‐7 and 35% on the RMDQ.

**TABLE 5:** Rates of clinically significant improvement and deterioration (complete‐case data) from pre‐treatment

	PHQ−9 (>=5, *N* = 274)		GAD−7 (>=5, *N* = 248)		RMDQ (>=8, *N* = 135)	
	Post‐Treatment	Follow‐up	Post‐Treatment	Follow‐up	Post‐Treatment	Follow‐up
Improvement (>=30%)	58.9% (126/214)	69.7% (131/188)	60.3% (117/194)	67.9% (114/168)	35.0% (41/117)	41.6% (32/77)
Deterioration (>=30%)	4.2% (9/214)	3.7% (7/188)	6.2% (12/194)	4.2% (7/168)	2.6% (3/117)	1.3% (1/77)

### 3.5 Secondary outcome measures

As seen in Table 4, the GEE analyses showed significant time effects on all of the secondary measures from pre‐treatment to post‐treatment and follow‐up (BPI: *F*_(2180)_ = 9.2, *p* < 0.001; CPAQ: F_(2234)_ = 36.4, *p* < 0.001; PSEQ: F_(2246)_ = 48.0, *p* < 0.001; TSK: F_(2218)_ = 48.2, *p* < 0.001). Changes were moderate on the CPAQ‐8, PSEQ and TSK at both post‐treatment and 3‐month follow‐up. Changes on BPI‐Severity were small at both post‐treatment and 3‐month follow‐up.

### 3.6 Changes in medication and health service use

Among the subsample of patients enrolled from January 2018 onward (*n* = 150), a significant decrease was observed in the proportion of patients who were taking prescription medication for pain or mental health (82.0%, *n* = 123 vs. 57.0%, *n* = 61; *ꭙ2* (1, *n* = 107) = 20.83, *p* < 0.001) and seeing a GP/nurse/medical specialist (54.0%, *n* = 81 vs 45.4%, *n* = 49; *ꭙ2* (1, *n* = 108, =4.65, *p* = 0.03) between pre‐treatment and 3‐month follow‐up. Changes in the proportion of patients seeing a psychiatrist (21.3%, *n* = 32 vs. 17.6%, *n* = 19; *ꭙ2* (1, *n* = 108) =0, *p* = 1.00) or psychologist/counsellor (28.7%, *n* = 43 vs. 33.3%, *n* = 36, *ꭙ2* (1, *n* = 108) =0.11, *p* = 0.75) were not significant between pre‐treatment and 3‐month follow‐up.

### 3.7 Predictors of treatment response

Results from the logistic regression models are presented in Table 6. Overall, 59% of patients with a pre‐treatment PHQ‐9 ≥ 5 improved at least 30% at post‐treatment, 60% of patients with pre‐treatment GAD‐7 ≥ 5 improved at least 30% and 35% of patients with pre‐treatment RMDQ ≥8 improved at least 30%. On the PHQ‐9: patients who were single/separated/widowed and had higher BPI scores were less likely to improve. Patients living in towns or villages and those with higher pre‐treatment CPAQ‐8 and PSEQ scores were more likely to improve. On the GAD‐7: patients with higher PHQ‐9 and BPI scores and taking prescription medication were less likely to improve. Patients who were living in small to medium cities or towns or villages and had higher CPAQ‐8 and PSEQ were more likely to improve. On the RMDQ: patients who were older, unemployed, had higher BPI or RMDQ, and were taking prescription medication were less likely to improve. Patients with higher CPAQ‐8 and PSEQ were more likely to improve. Despite these models reaching statistical significance, the coefficients of discrimination were low (D ≤ 0.07), indicating these logistic models were insufficient to predict treatment response.

**TABLE 6:** Univariate logistic models predicting >=30% improvement at post‐treatment (pooled imputations under MAR assumption)

Predictor	PHQ−9 (*N* = 274)				GAD−7 (*N* = 248)				RMDQ (*N* = 135)			
	*p*	D	Odds Ratio (95% CI)	Estimated Proportion Improving	*p*	D	Odds Ratio (95% CI)	Estimated Proportion Improving	*p*	D	Odds Ratio (95% CI)	Estimated Proportion Improving
Demographics												
Age	0.21	0.01	1.01 (0.99, 1.03)		0.20	0.01	1.01 (0.99, 1.04)		0.10	.01	0.98 (0.95, 1.00)	
*Sex*:		0.01				0.01				0.01		
Male				53.9%				53.6%				27.2%
Female	0.31		1.37 (0.74, 2.52)	61.5%	0.20		1.52 (0.80, 2.87)	63.7%	0.28		1.69 (0.66, 4.33)	38.7%
Relationship:		0.03				0.02				0		
In a relationship				65.7%				65.2%	0.53		0.77	37.8%
Single/Separated/Widowed	0.02		0.50 (0.28, 0.90)	49.0%	0.11		0.60 (0.33, 1.11)	53.1%			(0.34, 1.72)	31.9%
Education:		0.01				0.01				0.01		
High school or less				60.0%				60.0%				40.4%
Postschool qualification/Some university	0.68		0.87 (0.44, 1.70)	56.5%	0.97		0.99 (0.49, 1.99)	59.7%	0.83		0.90 (0.35, 2.29)	37.9%
Degree qualification	0.40		1.38 (0.65, 2.94)	67.5%	0.52		1.31 (0.58, 2.97)	66.3%	0.48		0.68 (0.23, 1.97)	31.5%
Employment:		0.03				0.02				0.06		
Full‐time			1.77 (0.51, 6.21)	64.8%				65.0%				52.1%
Part‐time or casual	0.37		0.62 (0.27, 1.46)	76.6%	0.75		1.22 (0.36, 4.16)	59.4%	0.60		0.71 (0.19, 2.60)	43.4%
Unemployed	0.28		0.58 (0.29, 1.19)	53.5%	0.24		0.58 (0.23, 1.44)	51.8%	0.05		0.22 (0.05, 0.95)	19.6%
Disability	0.14			51.8%	0.18		0.61 (0.30, 1.26)	53.1%	0.15		0.49 (0.19, 1.27)	34.8%
Location:		0.04				0.04				0.04		
Large City				51.7%				51.3%				35.7%
Small to Medium City	0.13		1.73 (0.85, 3.50)	65.0%	0.02		2.66 (1.16, 6.08)	73.7%	0.36		0.64 (0.24, 1.66)	26.1%
Town or Village	0.02		2.51 (1.18, 5.34)	72.9%	0.06		2.04 (0.96, 4.34)	68.2%	0.17		2.02 (0.74, 5.55)	52.9%
Farm	0.94		0.97 (0.39, 2.41)	50.9%	0.71		1.21 (0.44, 3.28)	55.9%	0.75		0.81 (0.22, 2.96)	31.0%
Pre‐treatment severity												
PHQ−9	0.83	0	0.99 (0.94, 1.05)		0.04	0.02	0.94 (0.90, 1.00)		0.41	0.01	0.97 (0.91, 1.04)	
GAD−7	0.20	0.01	0.97 (0.92, 1.02)		0.23	0.01	0.96 (0.90, 1.02)		0.31	0.01	0.96 (0.90, 1.03)	
BPI	0.09	0.01	0.85 (0.71, 1.03)		0.02	0.03	0.80 (0.66, 0.97)		0.04	0.04	0.75 (0.58, 0.98)	
CPAQ−8	0.01	0.03	1.05 (1.01, 1.09)		0.07	0.02	1.03 (1.00, 1.07)		0.01	0.07	1.08 (1.02, 1.14)	
PSEQ	0.01	0.03	1.03 (1.01, 1.05)		0.04	0.02	1.02 (1.00, 1.05)		0.06	0.04	1.03 (1.00, 1.07)	
TSK	0.54	0	1.01 (0.97, 1.06)		0.83	0	0.99 (0.95, 1.04)		0.48	0.01	0.98 (0.92, 1.04)	
RMDQ	NA	NA	NA		NA	NA	NA		0.02	0.05	0.89 (0.82, 0.98)	
Clinical Predictors												
Pain length (Years)	0.81	0	1.00 (0.96, 1.03)		0.20	0.01	1.03 (0.99, 1.07)		0.73	0	1.01 (0.96, 1.05)	
Number of pain sites	0.59	0	0.98 (0.90, 1.06)		0.60	0	0.98 (0.89, 1.07)		0.11	0.02	0.90 (0.80, 1.02)	
Prescription Medication:		0.01				0.02				0.03		
No				70.3%				76.0%				57.8%
Yes	0.19		0.58 (0.26, 1.31)	58.0%	0.07		0.44 (0.19, 1.05)	58.5%	0.06		0.36 (0.12, 1.03)	32.7%
Referral Type:		0.01				0.02				0		
Physician Referral				65.5%				66.0%				37.4%
Other Referral^a^	0.31		0.73 (0.40, 1.34)	58.0%	0.12		0.60 (0.32, 1.13)	54.0%	0.84		0.92 (0.41, 2.06)	35.4%
Other Source	0.20		0.61 (0.29, 1.30)	53.8%	0.92		1.04 (0.46, 2.38)	67.0%	0.78		1.18 (0.37, 3.80)	41.4%
Saw any Health Care Service		0.01				0				0		
No	0.29		0.65 (0.30, 1.44)	67.9%	0.67		0.86 (0.42, 1.75)	64.2%	0.86		0.87 (0.20, 3.81)	39.2%
Yes				58.1%				60.5%				36.0%

Note

PHQ‐9, Patient Health Questionnaire‐9; GAD‐7, Generalized Anxiety Disorder‐7; RMDQ, Roland Morris Disability Measure; BPI‐Severity, Brief Pain Inventory; CPAQ‐8, Chronic Pain Acceptance Questionnaire 8‐item; PSEQ, Pain Self‐Efficacy Questionnaire; TSK, TAMPA Scale of Kinesiophobia.

^a^ Other referrals include mental health professionals, bariatric clinic referral or family member/friend/employer. Other source includes printed materials, media or online search. Results were considered significant if *p* ≤ 0.1. D is the coefficient of discrimination.

## 4 DISCUSSION

The aim of this observational longitudinal study was to examine the effectiveness and acceptability of an Internet‐delivered cognitive behavioural PMP over a 5‐year period when provided as routine care in an online therapy clinic. This study also explored predictors of treatment response. Examination of an Internet‐delivered cognitive behavioural PMP in this setting is valuable, given that this is the context in which these emerging programmes intend to be used (Eccleston et al., 2020) and given that patients in routine care are known to present with greater symptom severity and diversity than patients in RCTs (Gibbons et al., 2013). The observational data from routine care are helpful, feasible, affordable complement to early phase RCT data (Thadhani, 2006), and specifically assists with understanding the effects of Internet‐delivered cognitive behavioural PMPs under different conditions.

The overall sample showed significant improvements for depression and anxiety at post‐treatment (depression: 35%; anxiety 37% reduction) and at 3‐month follow‐up (depression: 41%; anxiety: 41% reduction). Significant improvements were also found on secondary measures of pain severity, fear of movement and re‐injury and chronic pain acceptance among the overall sample. Furthermore, fewer patients reported taking medication and visiting a medical professional in the follow‐up period compared to pre‐treatment. High rates of treatment completion (79.5%) and the fact that most patients completed satisfaction measures and regarded the treatment as worth their time (96.2%, 203/211) suggest that Internet‐delivered cognitive behavioural PMPs are also an acceptable intervention. The results were comparable to previous controlled studies of the *Pain Course* (e.g. Dear et al., 2013, 2015, 2017), with our previous publication that reported the outcomes of the first 55 patients who enrolled in the *Pain Course* in the same routine online therapy clinic (Hadjistavropoulos et al., 2018), and with outcomes reported in more traditional low‐intensity cognitive behavioural PMPs (Carnes et al., 2012; Williams et al., 2012). This latter finding is encouraging since cognitive behavioural PMPs are variable in nature in terms of the duration of treatment (i.e. 3 weeks to 11 months; Buhrman et al., 2016), amount of therapist support (i.e. self‐directed to weekly telephone calls; Buhrman et al., 2016), as well as the extent to which various skills are emphasized (e.g. psychological, mind‐body, physical activity, lifestyle and pain education; see Carnes et al., 2012 and Eccleston et al., 2014 for a review). Carnes et al. (2012) found that longer courses of treatment did not result in better outcomes than shorter courses, and recommended that PMPs <8 weeks long should be seriously considered, which is consistent with the length of the intervention examined in this study.

An additional objective of this study was to identify characteristics of patients most likely to benefit from Internet‐delivered cognitive behavioural PMPs. A liberal *p*‐value of 0.1 was used to identify predictors that can provide valuable information for therapists and services. Predictors of treatment outcomes have previously been examined by Dear et al. (2016), using patients who volunteered for an RCT of the *Pain Course*. That trial failed to find any ‘particularly decisive or dominant predictors… that were common across time points or across the outcome domains’ (p. 2264). In the current study, there was somewhat greater consistency in some measures that predicted outcomes, but the magnitude of the prediction effects was small. In terms of notable patterns, low pain self‐efficacy and pain acceptance and high pain intensity at pre‐treatment were significant predictors of poorer treatment response for depression, anxiety and disability. Also notable was that taking prescription medication was predictive of poorer outcomes on anxiety and disability. While these variables were found to be consistent across our primary outcome measures, the coefficients of discrimination were small and the ability to predict treatment outcomes were weak. Consequently, the results should not be taken to mean that patients with these characteristics will not improve or should not be offered treatment. Instead, it would appear that there is a lower probability of improvement, which could be helpful for clinicians and services to be aware of when delivering these programmes. For example such information could guide clinicians to provide more support to such patients during these programmes, or to take other measures that might enhance the likelihood of positive treatment outcomes, such as coordinating care with patient’s medical practitioners or encouraging use of physical therapists alongside the programme. In general, the findings of this study are consistent with previous research which suggests that pain self‐efficacy plays a role in long‐term disability outcomes (Puschmann et al., 2020), and that higher levels of pain acceptance at pre‐treatment are associated with greater improvements in pain intensity and disability (Shaygan et al., 2018). Furthermore, lower baseline levels of pain and staying off opioid medications have also been associated with a more durable treatment response (Huffman et al., 2019). In the future, it may be worthwhile investigating if it would be helpful for clinicians to provide more support to patients who have low pain self‐efficacy, pain acceptance and or high pain intensity scores at pre‐treatment.

An important finding from the study was the observed growth in numbers of patients enrolling in the programme from 64 patients during the first full year to 133 patients in 2019. This represents a significant increase in demand, and is promising, as previous research suggests that Internet‐delivered cognitive behavioural PMPs contribute to considerable healthcare cost‐savings for each additional clinical outcome (i.e. depression, anxiety, disability and pain) that is reduced by 30% or greater (Dear et al., 2021).

Of interest from an implementation perspective, although initially we intended to offer support primarily by telephone, given challenges in scheduling telephone calls with patients, we began to ask patients about their preferences for support offered via telephone or email. This ultimately signalled patient preference for email over telephone support. Subsequently, patients were provided primarily email support and phone support was offered only when clinically indicated. In future research, it may be valuable to randomly assign patients to telephone or email support and assess the extent to which the two approaches differ. Our experience is that offering support via email is practically easier to implement as there is no need to schedule appointments. Emails also have the advantage that patients can reread previous messages from their therapists to review key points that might be missed or forgotten during a telephone conversation.

Overall, the study findings need to be considered in light of some limitations. First, as this study was conducted as part of routine care in an online therapy clinic, some challenges were encountered with data collection, resulting in some missing data at post‐treatment and follow‐up, although still within acceptable limits on depression and anxiety measures (22.8% missing post‐treatment and 33.1% at follow‐up). The RMDQ was not administered to the last 139 participants, and completion of secondary measures at 3‐month follow‐up was low (44%). There were inconsistencies in questions related to medication and health service use resulting in this data available on a subsample of patients (*n* = 150). Consistent with routine care research, patients accessed other services while completing the *Pain Course*. As with other studies of the *Pain Course* (e.g. Dear et al., 2013, 2015, 2017; Friesen et al., 2017; Hadjistavropoulos et al., 2018), our sample consisted predominantly of patients who identified as female, Caucasian and well‐educated, which may limit generalizability. However, at the same time, the current findings and previous findings (Dear et al., 2017) do not suggest these factors to be significant predictors of clinical outcomes.

## 5 CONCLUSIONS

The current study confirms the generalizability of past research on the acceptability and effectiveness of an Internet‐delivered cognitive behavioural PMP (i.e. the *Pain Course*) when offered as routine care by an online therapy clinic with a large help‐seeking sample. Predictors of poorer outcomes at post‐treatment included lower pain acceptance, lower pain self‐efficacy and higher pain intensity at pre‐treatment across anxiety, depression and disability, which potentially provides beneficial knowledge for clinicians providing Internet‐delivered cognitive behavioural PMPs. Future research that explores whether providing additional therapeutic support to patients who have low pain acceptance, low pain self‐efficacy and higher pain intensity at pre‐treatment could be worthwhile.

## CONFLICTS OF INTEREST

No authors have any conflicts of interest/disclosures to report. Dr. Heather Hadjistavropoulos declares funding as noted above. Dr. Blake Dear and Professor Nickolai Titov are developers of the *Pain Course*, but derive no personal or financial benefit from it. Professor Nickolai Titov and Dr. Blake Dear are funded by the Australian Government to develop and provide a free national online assessment and treatment service, the MindSpot Clinic (www.mindspot.org.au), for people with anxiety, depression and chronic pain.

## AUTHORS’ CONTRIBUTIONS

HDH designed the study, oversaw analysis and interpretation of data, and co‐drafted the article. VP and DT analysed the data and co‐drafted the article. AW and EK worked on analysis and interpretation of data and revising the paper for important intellectual content. MN assisted with design of study, data analysis and revising the paper for important intellectual content. LS, BD and NT assisted with design of the study and revised the paper for important intellectual content. All authors approved the final version of the paper.
